# Predicting Pathological Complete Response Following Neoadjuvant Therapy in Patients With Breast Cancer: Development of Machine Learning–Based Prediction Models in a Retrospective Study

**DOI:** 10.2196/64685

**Published:** 2025-07-18

**Authors:** Chun-Chi Lai, Cheng-Yu Chen, Tzu-Hao Chang

**Affiliations:** 1 Graduate Institute of Biomedical Informatics, College of Medical Science and Technology, Taipei Medical University, 9th Floor, 301 Yuantong Road, Zhonghe District, Taipei, Taiwan, 886 66202589 ext 10928; 2 Division of General Surgery, Department of Surgery, New Taipei Municipal TuCheng Hospital, New Taipei City, Taiwan; 3 Department of Medical Imaging, Taipei Medical University Hospital, Taipei, Taiwan; 4 Department of Radiology, School of Medicine, College of Medicine, Taipei Medical University, Taipei, Taiwan; 5 Research Center for Artificial Intelligence in Medicine, Taipei Medical University, Taipei, Taiwan; 6 Department of Radiology, National Defense Medical Center, Taipei, Taiwan; 7 Translational Imaging Research Center, Taipei Medical University Hospital, Taipei, Taiwan; 8 Clinical Big Data Research Center, Taipei Medical University Hospital, Taipei, Taiwan

**Keywords:** breast cancer, neoadjuvant therapy, pathological complete response, breast sonography, machine learning

## Abstract

**Background:**

Breast cancer is the most prevalent form of cancer worldwide, with 2.3 million new diagnoses in 2022. Recent advancements in treatment have led to a shift in the use of chemotherapy-targeted immunotherapy from a postoperative adjuvant to a preoperative neoadjuvant approach in select cases, resulting in enhanced survival outcomes. A pathological complete response (pCR) is a critical prognostic marker, with higher pCR rates linked to improved overall and disease-free survival.

**Objective:**

The objective of this study was to develop robust, machine learning–based prediction models for pCR following neoadjuvant therapy, leveraging clinical, laboratory, and imaging data.

**Methods:**

A retrospective cohort study was conducted using data from the Taipei Medical University Clinical Research Database from 2015 to 2022. Eligible patients were those with breast cancer who received neoadjuvant therapy followed by curative surgical resection. Machine learning models were developed using 3 distinct sets of variables. Model 1 included 14 clinical features such as age, height, weight, tumor stage, receptor status, tumor markers, and intrinsic subtype. Model 2 expanded on this by incorporating additional laboratory data and comorbidities (29 variables in total). Model 3 added breast sonography response data to the clinical variables in model 1. Algorithms including logistic regression, random forest, support vector machines, and extreme gradient boosting were used. Feature selection was performed using recursive feature elimination with cross-validation, and model performance was assessed using accuracy and area under the receiver operating characteristic curve (AUROC).

**Results:**

A total of 334 patients were analyzed, with 199 in the non-pCR group and 135 in the pCR group. The application of logistic regression with recursive feature elimination with cross-validation was found to demonstrate the optimal performance among the various algorithms that were evaluated in this study. Model 1 attained a mean accuracy of 0.66 (SD 0.02) and a mean AUROC of 0.73 (SD 0.01). The incorporation of laboratory data and comorbidities in model 2 did not yield significant enhancement, with a mean accuracy of 0.67 (SD 0.02) and a mean AUROC of 0.73 (SD 0.01). The incorporation of breast sonography response in model 3 led to a modest enhancement in predictive performance for the sonography group (accuracy 0.68; AUROC 0.60) in comparison to the nonsonography group (accuracy 0.66; AUROC 0.55). Despite the modest sample size (41 patients) of model 3, the integration of sonography data appeared to offer additional value in predicting pCR and warrants further investigation.

**Conclusions:**

This study suggests that incorporating breast sonography into models with clinical and laboratory data may modestly improve pCR prediction. It is important to note that the findings of this study are preliminary and require cautious interpretation. Further studies are required to validate this approach and support its integration into a machine learning–based clinical workflow.

## Introduction

### Breast Cancer and Treatment Strategies

Breast cancer is the most prevalent cancer worldwide, with approximately 2.3 million women diagnosed and 670,000 deaths reported in 2022 [1]. In Taiwan, the disease is also the most prevalent form of cancer among women, with an annual incidence of 15,000 new cases [2]. Treatment strategies for breast cancer are highly personalized, encompassing surgery, chemotherapy, targeted therapy, immunotherapy, hormone therapy, and radiotherapy [3,4]. Historically, the sequence of chemotherapy, targeted therapy, and immunotherapy was implemented as an adjuvant treatment following surgery. However, in certain circumstances, it is now used as neoadjuvant therapy, administered prior to surgery, to optimize outcomes [5].

### The Role and Advantages of Neoadjuvant Therapy

Neoadjuvant therapy confers a number of clinical advantages, including the potential to reduce the size of the tumor, thereby enabling breast-conserving surgery in patients who might otherwise require mastectomy. This approach also provides a critical window to monitor tumor regression and assess pathological responses, which are strongly correlated with improved overall survival and disease-free survival [6-8]. It is imperative to acknowledge that the efficacy of neoadjuvant therapy exhibits substantial intrinsic subtype variability, underscoring the necessity for subtype-specific therapeutic strategies.

Current guidelines recommend neoadjuvant therapy for patients with ≥ cT2 or cN(+) luminal-type breast cancer and ≥ cT1c, cN0 human epidermal growth factor receptor 2 (HER2)-positive or triple-negative breast cancer (TNBC) [9]. In the context of HER2-positive breast cancer, the Adjuvant Paclitaxel and Trastuzumab trial has demonstrated the efficacy of a de-escalated regimen of TH (paclitaxel and trastuzumab) for 12 cycles in achieving excellent outcomes for lower-risk patients [10]. In the context of TNBC, the incorporation of carboplatin into standard neoadjuvant chemotherapy has been shown to result in a substantial enhancement in pathological complete response (pCR) rates [11]. Moreover, the addition of pembrolizumab in conjunction with chemotherapy has been observed to further augment pCR rates and event-free survival, particularly in cases classified as high risk [12].

An accurate assessment of tumor response is imperative for the optimization of neoadjuvant therapy strategies. Previous studies have investigated a multitude of factors to predict pCR in patients with breast cancer undergoing neoadjuvant therapy [13]. Imaging modalities, such as breast sonography, are valuable for evaluating tumor size and margins, with a sensitivity of 61% and specificity of 78% [14]. Breast sonography plays a pivotal role in assessing response during treatment and complementing clinical examination, which remains a reliable and accessible method for evaluating palpable lesions. This dual approach enables the timely identification of nonresponders, who can then be spared the adverse effects of ineffective chemotherapy and redirected to alternative therapeutic strategies with potentially greater efficacy [15].

Despite the variability in pCR rates (10%‐60%) across different breast cancer intrinsic subtypes, accurate prediction of pCR using a combination of clinical features, imaging techniques, and pathology remains critical for tailoring treatment and improving outcomes [16,17].

### Study Objective

The objective of this study is to develop a user-friendly prediction model for pCR after neoadjuvant therapy by integrating clinical features, advanced machine learning techniques, and breast sonography data. The implementation of such a model has the potential to facilitate the early identification of nonresponders, thereby enabling timely therapeutic adjustments and averting the administration of ineffective treatments. The ultimate implementation of this model in clinical practice could enhance personalized treatment plans, improve patient outcomes, and optimize health care resource use.

## Methods

### Study Design and Data Source

This retrospective cohort study used data from the Taipei Medical University Clinical Research Database (TMU-CRD), an electronic medical record system established in 2015. The dataset used in this study was exclusively derived from the TMU-CRD, which consolidates data from 3 affiliated academic hospitals: Taipei Medical University Hospital, Wan Fang Hospital, and Shuang Ho Hospital. The database is managed and maintained by these academic centers, ensuring high data quality and reliability. The TMU-CRD encompasses both structured data, including patient demographics, International Classification of Diseases codes, laboratory results, treatment procedures, medication records, and cancer registry entries, as well as unstructured data, comprising physician notes, imaging reports, and pathological reports. As of 2024, the database encompasses data from 1998 to 2022 and contains the medical records of nearly 4.3 million patients across Taiwan.

### Patient Selection

The population of this study included patients diagnosed with breast cancer who underwent neoadjuvant therapy followed by curative-intent surgical resection. Patients were identified using a specific label in the cancer registry that indicated neoadjuvant treatment. Exclusion criteria encompassed instances where chemotherapy records were incomplete, where chemotherapy was not administered prior to surgery, and the presence of metastatic disease. Following the application of these criteria, a final cohort of 334 patients was identified for the purposes of analysis.

### Data Collection

A comprehensive dataset, encompassing clinical and imaging parameters, was meticulously collected to develop predictive models for pCR. The demographic variables included age, height, and weight, clinical tumor size, clinical TNM stage, hormone receptor (estrogen receptor [ER] or progesterone receptor [PR]) status, HER2 status, Ki-67 expression, breast cancer intrinsic subtype, Nottingham Bloom Richardson (NBR) grading, carcinoembryonic antigen (CEA) levels, carbohydrate antigen 15‐3 (CA15-3) levels, laboratory test results, and comorbidities. The intrinsic subtypes of breast cancer were categorized as hormone receptor-positive, HER2-positive, and triple-negative. The evaluation of imaging data, specifically breast sonography responses, was conducted based on Response Evaluation Criteria in Solid Tumors criteria and categorized into complete response, partial response, stable disease, or progression disease. The pathological response was classified as complete response (ypT0/Tis and ypN0) or residual disease.

The Allred scoring system was used to determine hormone receptor (ER or PR) status. The assessment of HER2 status was conducted through immunohistochemistry, and cases that yielded a score of 2+ underwent additional evaluation via fluorescence in situ hybridization. The TNM staging was determined according to the American Joint Committee on Cancer guidelines.

### Machine Learning Models

Three models were constructed for the development of machine learning models to predict pCR. Model 1 consisted of 14 clinical parameters, including age, height, weight, BMI, tumor size, clinical N stage, ER status, PR status, HER2 status, Ki-67 expression, NBR grading, CEA levels, CA15-3 levels, and breast cancer intrinsic subtype. Model 2 expanded upon model 1 by incorporating additional laboratory test results and comorbidities, resulting in a total of 29 variables. Model 3 included the parameters from model 1 and integrated breast sonography response data as an additional feature. Due to limited sonography data availability, this dataset included only 41 patients. Model 2 expanded upon model 1 by incorporating additional laboratory test results and comorbidities, resulting in a total of 29 variables.

### Statistical Analysis and Machine Learning Approach

Univariate analysis was performed using 2-tailed *t* tests and chi-square tests to identify significant differences between the pCR and non-pCR groups. Features that exhibited statistical significance (*P*<.05) and those that were supported by existing literature were selected for model construction. Missing values were addressed through the implementation of imputation methods, with mean imputation being applied to continuous variables and an additional category being created for categorical variables. With regard to missing values, we excluded variables for which more than 30% of data points were missing.

The performance of the model was evaluated using accuracy and the area under the receiver operating characteristic curve (AUROC). The AUROC is a metric that quantifies a model’s ability to distinguish between 2 classes (eg, pCR vs non-pCR) by plotting the true positive rate (sensitivity) against the false positive rate (1−specificity) at various thresholds. An AUROC value of 1.0 indicates perfect classification, while a value of 0.5 represents no better performance than random chance. The College of American Pathologists asserts that an area under the curve below 0.6 indicates inadequate discriminative ability, values between 0.6 and 0.75 suggest beneficial discrimination, and values above 0.75 reflect substantial clinical utility. It is therefore generally recommended that an AUROC of at least 0.6 is attained for a prognostic model if its use in clinical settings for the prediction of cancer outcomes is to be considered beneficial [18].

The 3 models were analyzed using the following algorithms: logistic regression (LR), random forest, support vector machine, and extreme gradient boosting. The feature selection process was optimized through the implementation of recursive feature elimination with cross-validation. The training and testing datasets were partitioned into 80% and 20% of the total data, respectively, with the exception of model 3, where leave-one-out cross-validation (LOO-CV) was used due to the limited sample size. To interpret the importance of breast sonography in model 3, Shapley Additive Explanations (SHAP) were used. The SHAP values were computed using the SHAP Python package in order to quantify the contribution of each feature to the model’s output. The mean absolute SHAP value was used to summarize feature importance, and the median rank of sonography response was calculated by comparing its importance across all features for each prediction. The performance of each model was evaluated using accuracy and AUROC metrics.

### Ethical Considerations

The study was reviewed and approved by the institutional review board (IRB) of Taipei Medical University, Taipei, Taiwan (IRB N202305036). The study entailed secondary analysis of deidentified data procured from the Taipei Medical University Clinical Research Database. In accordance with the regulations established by the IRB and the institutional policy, the requirement for informed consent was waived due to the fact that no personally identifiable information was accessed. The research was conducted in accordance with the ethical standards outlined in the Declaration of Helsinki. The participants did not receive any form of financial compensation, and no direct interaction occurred between the participants and the investigators. The maintenance of data privacy and confidentiality was strictly enforced throughout the study.

## Results

### Basic Characteristics

We identified 334 patients from the 3 hospital datasets. These patients were stratified into 2 groups based on their pCR status: the non-pCR group, comprising 199 (59.5%) individuals, and the pCR group, comprising 135 (40.4%) individuals. Univariate analysis revealed significant differences between the 2 groups in several clinical parameters, including tumor size, clinical T stage, ER status, PR status, HER2 status, Ki-67 expression, breast cancer intrinsic subtype, CA15-3 levels, and hemoglobin concentrations. However, no significant differences were observed in age, BMI, clinical N stage, overall clinical stage, NBR grading, or CEA levels (Table 1).

**Table 1.** Demographic data according to pCR^a^ (N=334).

		Non-pCR (n=199)	pCR (n=135)	*P* value^b^
Age (years), mean (SD)		53.6 (11.5)	52.4 (10.5)	.35
Height (cm), mean (SD)		157.1 (5.6)	157.1 (6.1)	.93
Weight (kg), mean (SD)		60.2 (10.7)	60.1 (11.7)	.90
BMI, mean (SD)		24.4 (4.4)	24.4 (4.8)	.93
Clinical tumor size (mm)				.01
	Mean (SD)	47.9 (26.5)	40.4 (22.8)	
	Missing, n (%)	4 (2)	7 (5.1)	
Clinical T stage, n (%)				.01
	T1	16 (8)	13 (9.6)	
	T2	100 (50.3)	88 (65.2)	
	T3	53 (26.6)	18 (13.3)	
	T4	30 (15.1)	16 (11.9)	
Clinical N stage, n (%)				.29
	N0	42 (21.1)	39 (28.9)	
	N1	126 (63.3)	73 (54.1)	
	N2	22 (11.1)	19 (14.1)	
	N3	9 (4.5)	6 (4.4)	
Stage, n (%)				.15
	1	4 (2)	4 (3)	
	2	108 (54.3)	86 (63.7)	
	3	87 (43.7)	45 (33.3)	
ER^c^, n (%)				<.001
	Positive	134 (67.3)	61 (45.2)	
	Negative	65 (32.7)	74 (54.8)	
PR^d^, n (%)				<.001
	Positive	117 (58.8)	44 (32.6)	
	Negative	82 (41.2)	91 (67.4)	
HER2^e^, n (%)				<.001
	Positive	66 (33.2)	89 (65.9)	
	Negative	133 (66.8)	46 (34.1)	
Ki-67				<.001
	Mean (SD)	39 (22.6)	49.1 (22.6)	
	Missing, n (%)	18 (9)	10 (7.4)	
Subtype, n (%)				<.001
	HR^f^ (+)	100 (50.3)	21 (15.6)	
	HER2 (+)	66 (33.2)	89 (65.9)	
	TNBC^g^	33 (16.6)	25 (18.5)	
NBR^h^, n (%)				.25
	0	4 (2)	0 (0)	
	1	79 (39.7)	56 (41.5)	
	2	40 (20.1)	26 (19.5)	
	Missing	76 (38.1)	53 (39.3)	
CEA^i^				.12
	Mean (SD)	10.7 (44.6)	4.2 (7.3)	
	Missing, n (%)	29 (14.5)	16 (11.8)	
CA15-3^j^				.04
	Mean (SD)	21.3 (37.8)	14 (9.7)	
	Missing, n (%)	32 (16)	18 (13.3)	
WBC^k^				.34
	Mean (SD)	7.12 (2.0)	7.34 (2.2)	
	Missing, n (%)	36 (18.1)	16 (11.8)	
Hemoglobin				<.001
	Mean (SD)	12.7 (1.7)	13.2 (1.3)	
	Missing, n (%)	35 (17.6)	16 (11.8)	
Platelet				.93
	Mean (SD)	268.4 (77.2)	269.2 (62.6)	
	Missing, n (%)	38 (19.1)	16 (11.8)	
Lymphocyte				.99
	Mean (SD)	27.0 (7.7)	27.0 (9.5)	
	Missing, n (%)	55 (27.6)	22 (16.2)	
Neutrophil				.80
	Mean (SD)	63.3 (8.6)	63.6 (10.7)	
	Missing, n (%)	55 (27.6)	22 (16.2)	
BUN^l^				.92
	Mean (SD)	12.7 (5.4)	12.8 (4.1)	
	Missing, n (%)	26 (13.1)	18 (13.3)	
Creatinine				.51
	Mean (SD)	0.72 (0.5)	0.68 (0.2)	
	Missing, n (%)	19 (9.5)	14 (10.3)	
GOT^m^				.89
	Mean (SD)	22.4 (11.4)	22.6 (11.8)	
	Missing, n (%)	40 (20.1)	19 (14.1)	
GPT^n^				.59
	Mean (SD)	21.6 (19.1)	22.8 (21.2)	
	Missing, n (%)	32 (16.1)	14 (10.3)	
Diabetes, n (%)				.89
	Yes	10 (5)	7 (5.2)	
	No	170 (85.4)	127 (94.1)	
	Missing	19 (9.5)	1 (0.7)	
Hypertension, n (%)				.19
	Yes	23 (11.6)	11 (8.1)	
	No	157 (78.9)	123 (91.1)	
	Missing	19 (9.5)	1 (0.7)	
Heart disease, n (%)				.83
	Yes	1 (0.5)	1 (0.7)	
	No	179 (99)	133 (98.5)	
	Missing	1 (0.5)	1 (0.7)	
Kidney disease, n (%)				.08
	Yes	4 (2)	0 (0)	
	No	176 (88.4)	134 (99.3)	
	Missing	19 (9.5)	1 (0.7)	
Asthma, n (%)				.83
	Yes	1 (0.5)	1 (0.7)	
	No	179 (89.9)	133 (98.5)	
	Missing	19 (9.5)	1 (0.7)	
Hepatitis B, n (%)				.91
	Yes	5 (2.5)	4 (3)	
	No	175 (88)	130 (96.3)	
	Missing	19 (9.5)	1 (0.7)	

a pCR: pathological complete response.

b *P* values are based on univariate analysis results using 2-tailed *t* tests and chi-square tests.

c ER: estrogen receptor.

d PR: progesterone receptor.

e HER2: human epidermal growth factor receptor 2.

f HR: hormone receptor.

g TNBC: triple-negative breast cancer.

h NBR: Nottingham Bloom Richardson.

i CEA: carcinoembryonic antigen.

j CA15-3: carbohydrate antigen 15-3.

k WBC: white blood cell.

l BUN: blood urea nitrogen.

m GOT: glutamic oxaloacetic transaminase.

n GPT: glutamic pyruvic transaminase.

Although breast sonography response did not achieve statistical significance in univariate analysis, a trend toward a higher pCR rate was observed in patients demonstrating superior sonography response (Table 2).

**Table 2.** Breast sonography responses with complete response and partial response are more likely to predict a pathological complete response (pCR) in final pathological result.

Sonography response	Non-pCR (n=28), n (%)	pCR (n=13), n (%)
Complete response	2 (7.1)	3 (23.1)
Partial response	13 (46.4)	8 (61.5)
Stable disease	12 (42.9)	2 (15.4)
Progression disease	1 (3.6)	0 (0)

### Machine Learning Features and Algorithms

The model 1 was analyzed using 6 machine learning algorithms, with LR incorporating recursive feature elimination and cross-validation demonstrating the best performance, yielding a mean accuracy of 0.66 (SD 0.02) and a mean AUROC of 0.73 (SD 0.01; Table 3).

**Table 3.** Performance of the predication model for models 1 and 2.

Algorithm	Accuracy, mean (SD)	AUROC^a^, mean (SD)
Model 1		
RF^b^	0.60 (0.02)	0.62 (0.04)
LR^c^	0.66 (0.01)	0.72 (0.02)
SVM^d^	0.60 (0.02)	0.64 (0.04)
XGB^e^	0.63 (0.02)	0.67 (0.03)
LR with RFECV^f^	*0.66 (0.02)^g^*	*0.73 (0.01)*
XGB with RFECV	0.65 (0.02)	0.70 (0.01)
Model 2		
RF	0.61 (0.02)	0.64 (0.03)
LR	0.66 (0.02)	0.72 (0.02)
SVM	0.59 (0.01)	0.59 (0.05)
XGB	0.64 (0.01)	0.68 (0.02)
LR with RFECV	*0.67 (0.01)*	*0.73 (0.01)*
XGB with RFECV	0.66 (0.03)	0.72 (0.01)

a AUROC: area under the receiver operating characteristic curve.

b RF: random forest.

c LR: logistic regression.

d SVM: support vector machine.

e XGB: extreme gradient boosting.

f RFECV: recursive feature elimination with cross-validation.

g The values in italics format indicate that LR with RFECV demonstrated the best performance among all evaluated algorithms.

In model 2, additional variables, including laboratory test results and comorbidities, were integrated with the clinical parameters from model 1, resulting in a total of 29 variables. However, the inclusion of these additional variables did not significantly improve predictive performance, with a mean accuracy of 0.67 (SD 0.02) and a mean AUROC of 0.73 (SD 0.01; Table 3).

**Figure 1.**
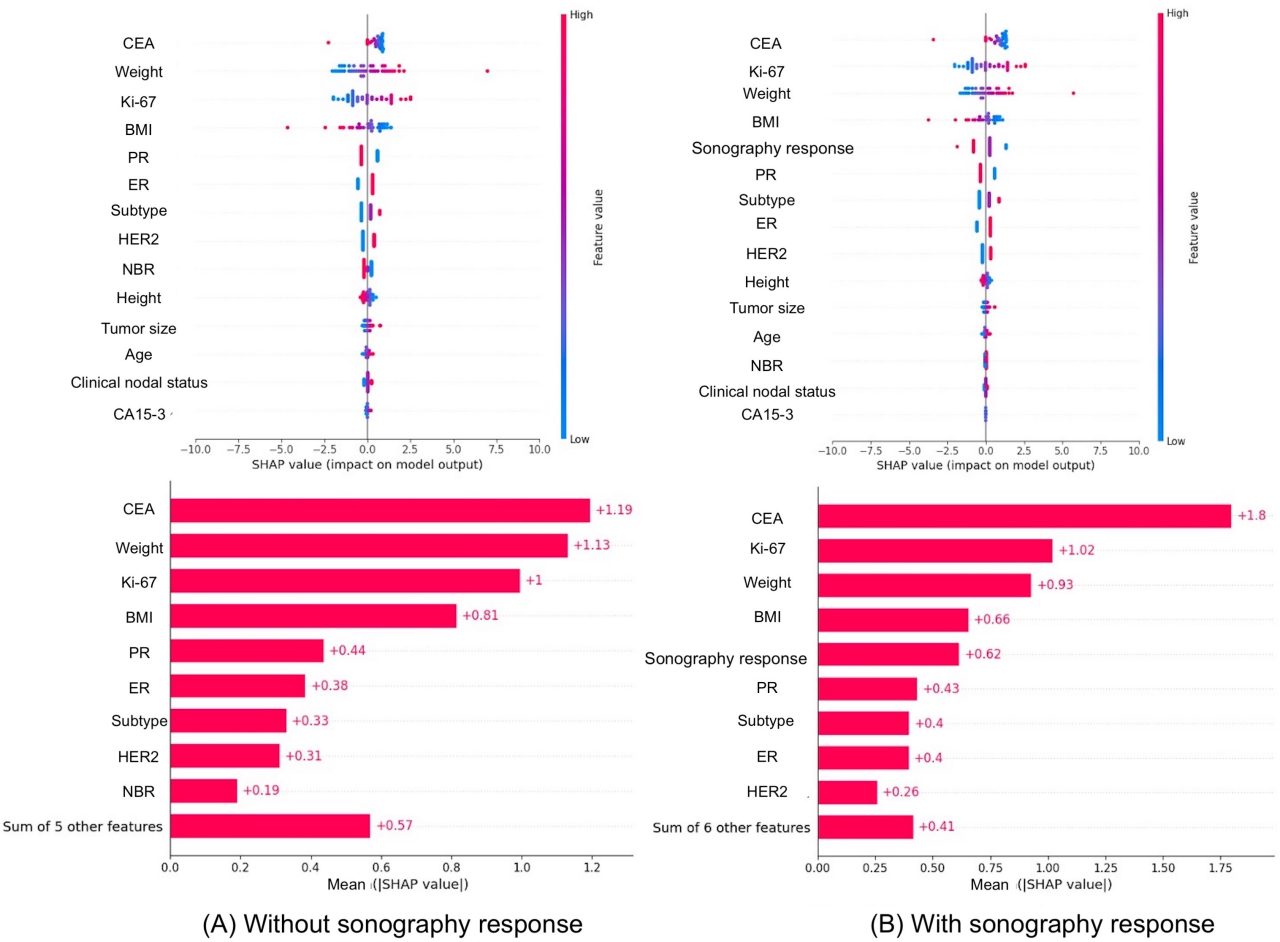
SHAP value for (A) without or (B) with breast sonography. CA15-3: carbohydrate antigen 15‐3; CEA: carcinoembryonic antigen; ER: estrogen receptor; HER2: human epidermal growth factor receptor 2; NBR: Nottingham Bloom Richardson; PR: progesterone receptor; SHAP: Shapley Additive Explanations.

**Figure 2.**
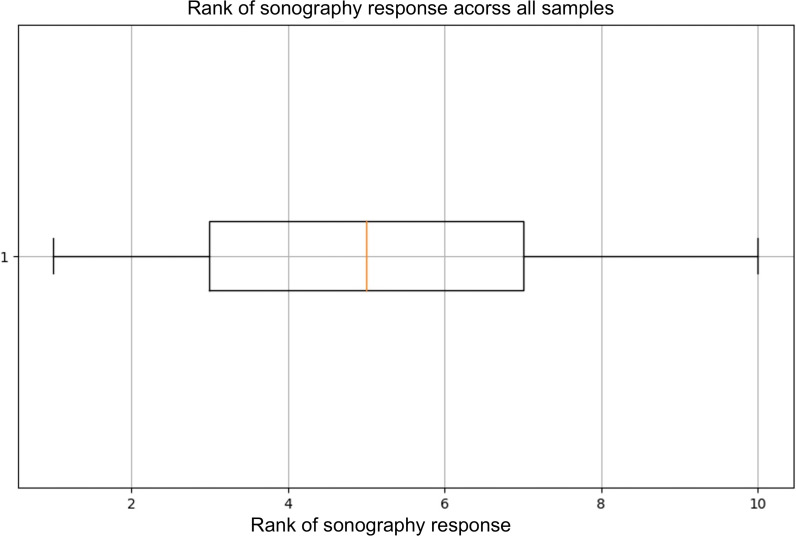
Boxplot illustrating the ranking of sonography response across all samples in model 3. The median rank of sonography response was 5 (IQR 3-7).

In model 3, the incorporation of breast sonography response data into the variables from the first model resulted in a dataset comprising 15 variables. Due to the limited sample size, the analysis used the LR algorithm with LOO-CV. The findings demonstrated that the incorporation of sonography response enhanced the predictive accuracy of the model, with an accuracy of 0.68 and an AUROC of 0.60 for the sonography group, as opposed to an accuracy of 0.66 and an AUROC of 0.55 for the nonsonography group. Furthermore, a SHAP value for sonography response was 0.62 (Figure 1), and the median rank of sonography response was 5 (IQR 3-7) among all samples in model 3 (Figure 2).

**Figure 3.**
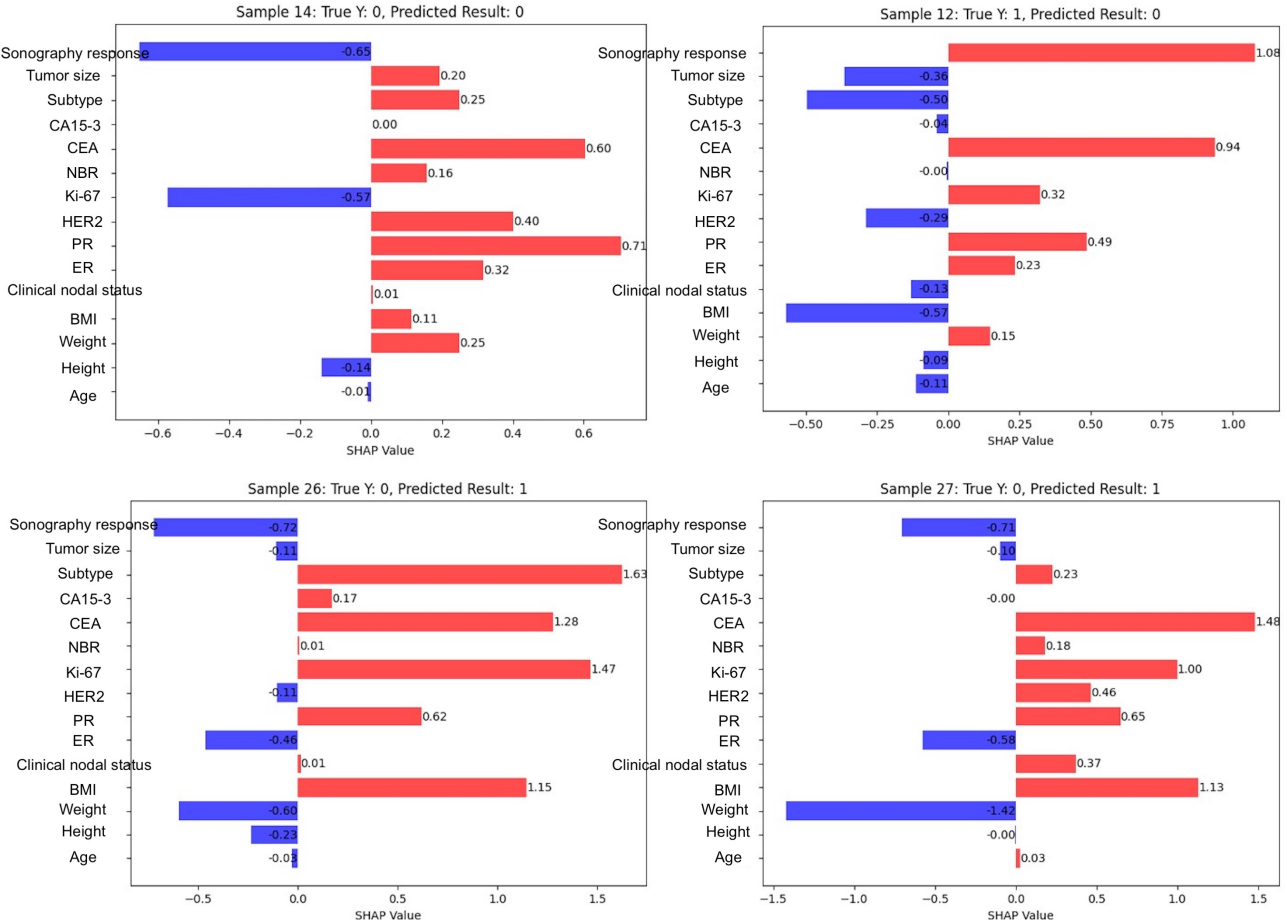
Selected samples from model 3 highlighting the significance of sonography response in prediction accuracy. In sample 14, the true and predicted results were non-pCR, but most variables incorrectly predicted pCR—except for the sonography response, which correctly indicated non-pCR. Similarly, in samples 12, 26, and 27, the predicted results were opposite to the actual outcomes; yet, the sonography response correctly aligned with the true results. CA15-3: carbohydrate antigen 15‐3; CEA: carcinoembryonic antigen; ER: estrogen receptor; HER2: human epidermal growth factor receptor 2; NBR: Nottingham Bloom Richardson; pCR: pathological complete response; PR: progesterone receptor; SHAP: Shapley Additive Explanations.

Subsequent analysis of specific samples in model 3 was undertaken to investigate the value of sonography response (Figure 3). In sample 14, the predicted and true results were non-pCR; however, most variables predicted pCR, with only the sonography response correctly indicating non-pCR, aligning with the final result. A similar pattern was observed in samples 12, 26, and 27, where the predicted results were incongruent with the true outcomes; nevertheless, the sonography response accurately reflected the actual results.

## Discussion

The objective of this study was to develop machine learning–based models to predict pCR in patients with breast cancer receiving neoadjuvant therapy, using clinical, laboratory, and imaging data. Three models were subjected to a rigorous testing process, and their predictive performance was thoroughly evaluated.

### Principal Findings

Model 1 incorporated clinical parameters alone, yielding an AUROC of 0.73. Model 2, which added laboratory data and comorbidities, demonstrated no significant improvement over model 1. Model 3, which incorporated a further integration of breast sonography response, demonstrated a modest enhancement in its predictive performance. This enhancement was characterized by an increase in AUROC from 0.55 to 0.60 and an increase in accuracy from 0.66 to 0.68. Furthermore, SHAP analysis indicated that sonography response contributed significantly to the model, ranking fifth among all the features considered. In some cases, sonography response aligned with true outcomes, despite the failure of other variables to do so. These findings indicate the potential value of incorporating imaging data into prediction models to enhance individualized treatment strategies.

### Comparison to Prior Work

The predictive performance of model 1 is consistent with that reported in previous studies, which used clinical parameters alone and reported AUROCs ranging from 0.65 to 0.77 [13,19-21]. The limited incremental value observed in model 2 is consistent with the findings in the literature that laboratory and comorbidity variables may not independently enhance prediction unless integrated with molecular or genetic data [21]. This study’s finding that breast sonography provides added predictive value is consistent with previous evidence highlighting the clinical utility of imaging in assessing tumor response, as demonstrated in model 3.

The observed pCR rates for the different intrinsic subtypes (HER2-positive, TNBC, and hormone receptor–positive) also reflect established trends in neoadjuvant therapy responsiveness [12,22-25].

### Challenges With Small Sample Size and Machine Learning Approaches

Despite extensive data mining, only 41 cases with sonography response data were available for model 3, thereby limiting the sample size and potentially increasing the risk of overfitting. To address this issue, we used an LR model with LOO-CV, a method that has been demonstrated to be particularly effective when dealing with small datasets. Initially, algorithms such as support vector machines (and decision trees were considered; however, imbalances in the dataset led to classification bias, which resulted in the adoption of more straightforward LR models. It is recommended that future research endeavors place a priority on the acquisition of more substantial datasets with a view to enhancing the model’s robustness and mitigating the risk of overfitting [26,27].

### Clinical Implications

**Figure 4.**
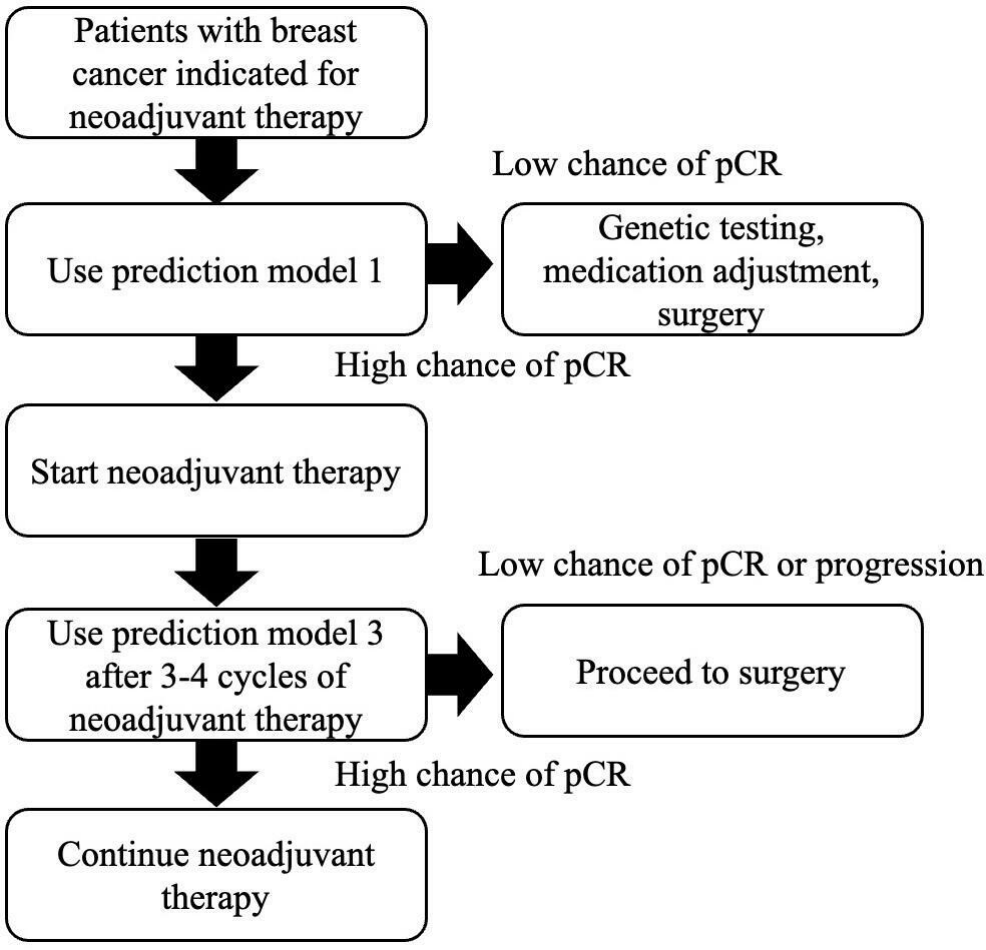
Conceptual flowchart outlining a potential application of the prediction model to support clinical decision-making. This framework is intended for illustrative purposes only and does not represent a validated clinical tool. pCR: pathological complete response.

The predictive models developed in this study have practical applications in clinical decision-making. Specifically, model 1, which uses only baseline clinical data, facilitates initial treatment planning by predicting the likelihood of pCR subsequent to a decision to proceed with neoadjuvant therapy. The third model, which incorporates midtreatment sonography response, enables dynamic reassessment of pCR probability, facilitating timely adjustments to therapeutic strategies, such as switching to alternative treatments or surgical interventions. The integration of machine learning into standard clinical workflows may offer potential benefits for improving outcomes in breast cancer care (Figure 4). Given the small sample and lack of validation, the prediction model has not reached clinical maturity, and it is therefore not prudent to make recommendations regarding its application.

### Strengths and Limitations

A significant strength of this study is its multimodal approach, integrating clinical, laboratory, and imaging data within machine learning models. The use of SHAP values enhances interpretability and facilitates the identification of relevant predictors, including sonography response.

However, it is important to note several limitations of this study. First, the sample size for model 3 was limited (n=41), thereby restricting statistical power and increasing the risk of overfitting. To mitigate this issue, the LOO-CV and LR methods were applied. Second, the data extracted from semistructured reports may have been subject to inconsistencies. Despite the evident benefits of text mining in enhancing the quality of the results, the use of standardized data collection methods in future studies would be a preferred approach. Third, the models did not include genetic or molecular biomarkers, which may limit their predictive performance. Fourth, as a retrospective, single-center study, there is a risk of selection bias and limited generalizability. Variables with over 30% missing data were excluded, a process that potentially resulted in the omission of important predictors. Furthermore, the absence of external validation of the model is a notable shortcoming.

### Future Directions

Future research endeavors should explore the integration of genomic, molecular, and radiomic data to further refine prediction models. Conducting prospective, multi-institutional studies with larger and more diverse patient cohorts is imperative to validate findings and support generalizability. Standardizing imaging report formats and leveraging natural language processing tools may also enhance data quality for imaging-based predictors. Consequently, these advancements have the potential to facilitate the development of robust clinical decision-support tools, thereby optimizing neoadjuvant treatment planning in breast cancer care.

### Conclusions

This study provides preliminary insight into the potential role of breast sonography response data in improving pCR prediction for patients with breast cancer receiving neoadjuvant therapy. The clinical and laboratory parameters that were provided served as a useful foundation for the models, and the addition of imaging data appeared to offer modest improvements in predictive accuracy. However, given the relatively small sample size, these findings should be interpreted with caution. The incorporation of imaging data into clinical workflows could potentially represent a valuable step toward the development of more personalized treatment approaches.

## Abbreviations

AUROC: area under the receiver operating characteristic curve
CA15-3: carbohydrate antigen 15‐3
CEA: carcinoembryonic antigen
ER: estrogen receptor
HER2: human epidermal growth factor receptor 2
IRB: institutional review board
LOO-CV: leave-one-out cross-validation
LR: logistic regression
NBR: Nottingham Bloom Richardson
pCR: pathological complete response
PR: progesterone receptor
SHAP: Shapley Additive Explanations
TMU-CRD: Taipei Medical University Clinical Research Database
TNBC: triple-negative breast cancer
